# Accurate Measurement Calculation Method for Interferometric Radar Altimeter-Based Terrain Referenced Navigation †

**DOI:** 10.3390/s19071688

**Published:** 2019-04-09

**Authors:** Juhyun Oh, Chang-Ky Sung, Jungshin Lee, Sang Woo Lee, Sang Jeong Lee, Myeong-Jong Yu

**Affiliations:** 1 Agency for Defense Development, Yuseong P.O.Box 35, Daejeon 34186, Korea; einstein@add.re.kr (C.-K.S.); jslee0534@add.re.kr (J.L.); 245489@add.re.kr (S.W.L.); mjyu@add.re.kr (M.-J.Y.); 2 Chungnam National University, 99 Daehak-ro, Yuseong-gu, Daejeon 34134, Korea; eesjl@cnu.ac.kr

**Keywords:** terrain referenced navigation, interferometric radar altimeter, flight test

## Abstract

In order to improve the performance of Terrain Referenced Navigation (TRN), an Interferometric Radar Altimeter (IRA) has been developed as a more accurate altimeter. The IRA outputs not only the relative distance (slant range, *R*) but also the cross-track angle (look angle, *θ*) of the closest point on the zero Doppler line by using the principle of interferometry and two or more antennas. To perform TRN using the IRA, the 3D relative position of the closest point should be calculated. There is a formula to calculate the relative position of the closest point using the Euler angles. However, in an actual flight environment in which the influence of wind exists, the angle of attack, the side slip angle and “the effective look angle” should be used rather than the Euler angles. In this paper, a new formula for calculating the relative position of the closest point is proposed and mathematically derived. The proposed formula was verified with real data from actual flight. The flight test results show that the positions of the closest points calculated using the conventional method and the proposed method are different because of the wind effect. The TRN simulation results indicate that the proposed formula calculates the closest points more accurately than the conventional formula.

## 1. Introduction

Terrain Referenced Navigation (TRN) is a technique for estimating the position of an aircraft by comparing the Digital Elevation Model (DEM) with the altitude of the terrain measured by an altimeter. This technique is used to correct the position error of the inertial navigation system, which increases as the navigation time becomes longer. The Global Navigation Satellite System (GNSS) is more accurate than TRN for correction of position error of the inertial navigation system and is used in various fields. However, GNSS has the disadvantage that it cannot be used if it receives a hostile jamming signal. On the other hand, TRN has the advantage of being able to operate normally regardless of hostile jamming signals. Because of this advantage, TRN is applied to various weapon systems such as Tomahawk and TAURUS missiles [1,2]. 

According to previous research results, the fundamental performance limitation of TRN is the Radar Altimeter (RA) beam width problem [3]. TRN expects an RA to measure a relative distance to a terrain point directly below the vehicle. However, RA measures the relative distance to the closest point in the beam footprint. The difference between the two altitudes is the RA error, which causes performance degradation of TRN. The smaller the RA beam width, the better the accuracy of TRN. There are methods of reducing the RA beam width problem by lowering the flight altitude as much as possible or making the angle of the beam width smaller by increasing the frequency of the radio wave used in the RA. However, these methods cannot be applied to objects flying at high altitude. 

To overcome these problems, an Interferometric Radar Altimeter (IRA) has been developed as a more accurate altimeter [4]. The IRA outputs not only the relative distance (slant range, *R*) but also the cross-track angle (look angle, *θ*) of the closest point on the zero Doppler line by using the principle of interferometry and two or more antennas. Since the IRA provides a three-dimensional measurement of the closest point, the beam width problem is no longer a problem.

In the 2000s, Honeywell developed a TRN system using an IRA called Precision Terrain Aided Navigation (PTAN). The published brochure states that PTAN has a position accuracy of about 3 m at altitudes up to about 1.5 km and about 30 m accuracy at altitudes up to about 9 km [5]. However, detailed papers or materials on PTAN have not been disclosed. Based on the findings on the IRA [4,6,7], many studies on TRN using IRA [8,9,10,11] have been conducted.

Recently, with Hanwha Systems, we developed a TRN system using IRA. The IRA was designed with reference to Raney’s paper [4]. We carried out several flight tests with the developed TRN system. To perform TRN using the IRA, the 3D relative position of the closest point should be calculated. In previous research, the relative position measurement equation for the closest point was presented [8]. However, when the equation was applied to an actual aircraft loading flight test, the altitude error was larger than expected. Analysis of the altitude error showed that the relative position of the closest point must be calculated using the angle of attack, the side slip angle and “the effective look angle” rather than the Euler angles, in an actual flight environment in which the influence of wind exists. 

This paper is an extension of work originally presented in reference [12]. In this paper, we propose a new relative position measurement equation that can be used in real flight environments where wind effects exist. We also prove the equation mathematically. To obtain real data for the TRN simulation, we carried out a flight test and analyzed the results. We evaluated the performance of the proposed equation and conventional equation by TRN simulation.

This paper is organized as follows. Section 2 describes differences between TRN using RA and TRN using IRA. In Section 3, the proposed relative position measurement equation for the closest point will be introduced after explanations of the existing relative position measurement equations. Section 4 presents performance evaluation of the proposed relative position measurement equation. The conclusion follows in Section 5.

## 2. Comparison of TRN Based on RA and IRA

### 2.1. TRN Using RA

**Figure 1:**
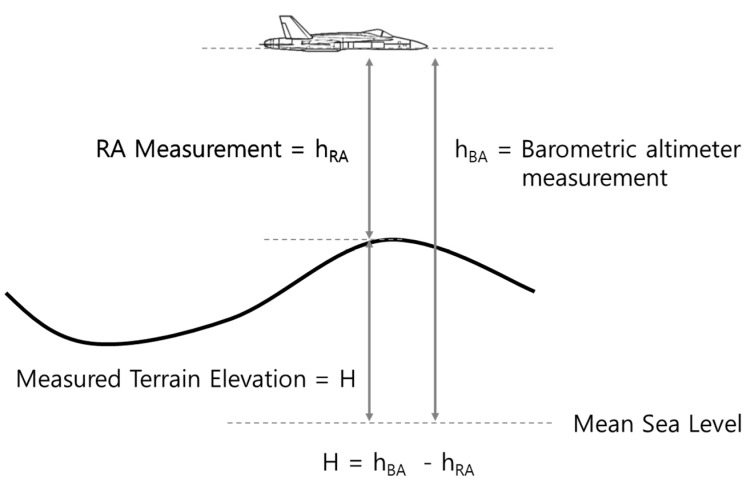
Measurement of TRN using RA.

**Figure 2:**
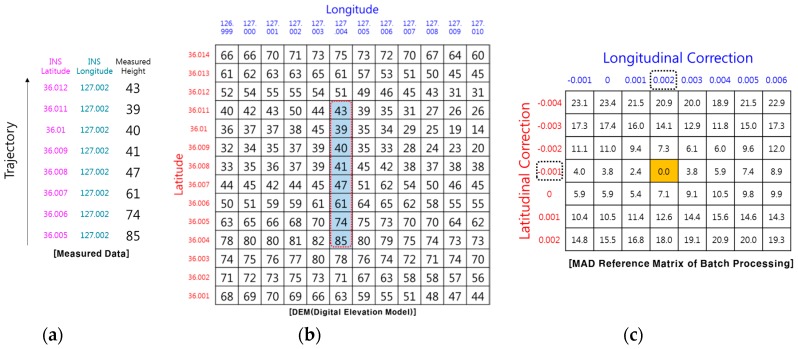
Example of TRN using RA (ideal case): (**a**) Measured terrain profile; (**b**) DEM; (**c**) position correction value.

Figure 1 and Figure 2 show examples of how TRN using RA finds its position. The TRN algorithm used in the example is a batch processing algorithm known as TERCOM [1]. Measuring the barometric altitude and RA altitude while the aircraft is flying along the trajectory, TERCOM can use the difference between the two altitudes to obtain the elevation of the ground directly below the aircraft (Figure 1). The latitude and longitude of the inertial navigation system are stored together with the elevation of the measured terrain as shown in Figure 2a. The DEM is embedded in the TRN system of the aircraft, so it has altitude information in terms of latitude and longitude for the trajectory as shown in Figure 2b. When the correction period of TRN is reached, it is possible to find the matching reference elevation set by comparing the measured terrain elevation set (Figure 2a) with the DEM (Figure 2b). Figure 2c shows the Mean Absolute Difference (MAD) reference matrix of the batch processing algorithm. The MAD algorithm which is used for correlating the measured terrain elevation file with each down track column of the reference matrix, is defined as follows [1].

(1) $$MAD_{i,j}=\frac{1}{M}\overset{M}{\underset{k=1}{\sum }}\left|h_{measured,k}-h_{DB}\left(\hat{\lambda }+i\Delta \lambda ,\hat{\varphi }+j\Delta \varphi \right)\right|$$

where *M* is the number of samples in the measured terrain elevation file. $h_{measured,k}$ and $h_{DB}$ are the terrain measurement and database value. $\hat{\lambda }$ and $\hat{\varphi }$ are estimated longitude and latitude and Δλ, Δφ are the longitudinal and latitudinal cell size of the reference matrix. *i* and *j* are the column and row index for the reference matrix.

The position correction value can be obtained by selecting the minimum MAD cell from the reference matrix. In the case, the position correction value is −0.001° in the latitude direction and +0.002° in the longitude direction.

**Figure 3:**
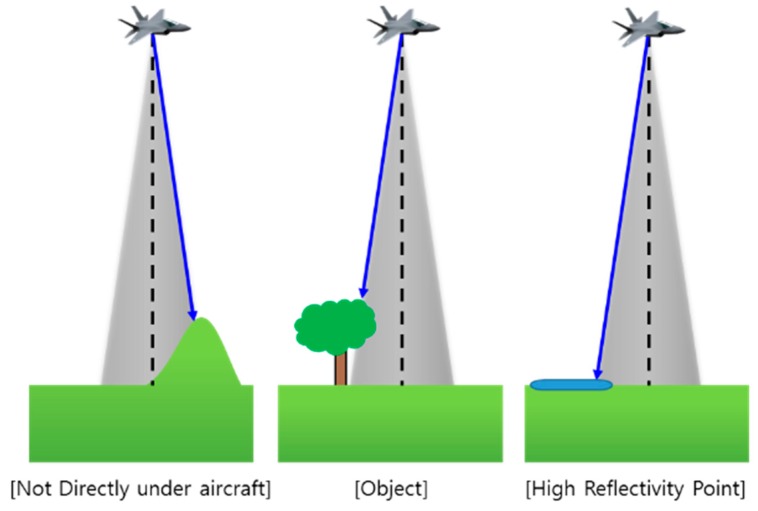
Error examples of RA [12].

**Figure 4:**
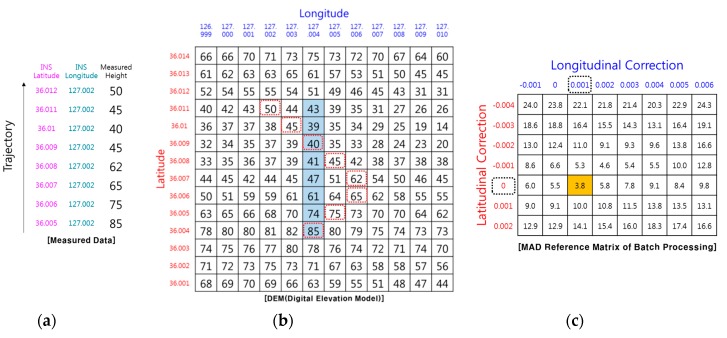
Example of TRN using RA (real case): (**a**) Measured terrain profile; (**b**) DEM; (**c**) position correction value.

In drawing Figure 2, it was assumed that the RA can measure the relative altitude of the terrain in the direct downward direction. However, the actual RA cannot always measure the relative altitude to the terrain directly below the aircraft because the actual RA outputs the relative distance to the closest point among points in the beam footprint. As shown in Figure 3, if there are other peaks in the radar beam footprint, objects such as trees or buildings, or objects with high reflectivity, such as lakes or oceans, the RA will measure the relative distance from the antenna to those points (blue arrow), and will not measure to a point directly below the aircraft (black dash line). If the media of the ground are all the same, the closest points measured by RA will be in peaks or ridges in the radar beam footprint. In Figure 4, the peaks on the DEM are marked with a dashed red box, and the altitude will be measured when the aircraft follows its trajectory. In the case of Figure 4, correction values of 0.000° in the latitude direction and 0.001° in the longitude direction can be obtained. However, these correction values are different from the correction values shown in Figure 2. In other words, the RA error generates a TRN error. In order to reduce the RA error, there is a method of reducing the beam width of the RA by lowering the flight altitude as much as possible or making the angle of the beam width smaller by increasing the frequency of the radio wave used in the RA. However, even if these methods are used, the RA error does not fundamentally disappear, and the fact that TRN error exists does not change. 

### 2.2. TRN Using IRA

**Figure 5:**
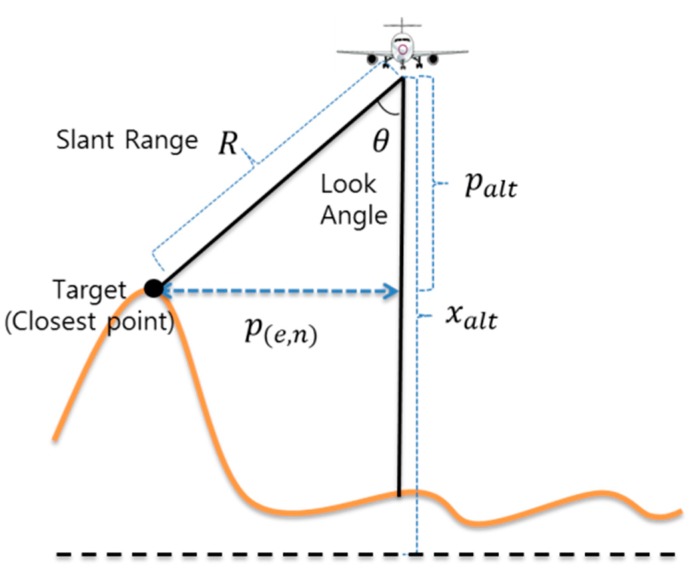
Measurement for TRN using IRA [8,12].

In order to overcome the disadvantages of RA described above, an IRA was developed [4]. The IRA outputs the slant range (*R*) to the closest point in the signal reflected from the zero Doppler region (line) and the look angle (*θ*) in the direction of the transverse axis (Figure 5). The IRA uses pulse compressing to select signals whose Doppler value is zero. In order to obtain the angle of the first returning signal, two or more antennas are placed in the transverse direction of the flight vehicle, and an interferometry method is used [13]. Since the IRA outputs the look angle, unavailable in RA, it is possible to more precisely measure the relative horizontal and vertical positions from the aircraft to the closest point. 

**Figure 6:**
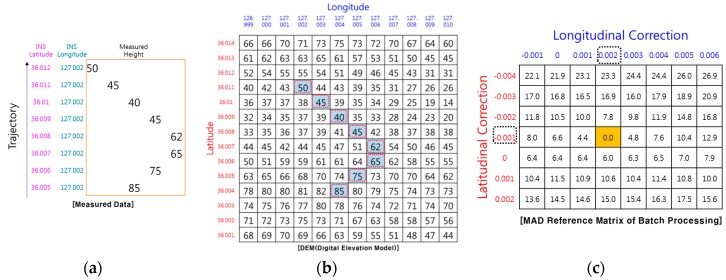
Example of TRN using IRA: (**a**) Measured terrain profile; (**b**) DEM; (**c**) position correction value.

An example of TRN using IRA is shown in Figure 6. This IRA can be used to determine not only the relative altitude of the closest point but also the horizontal relative position. In this case, MAD is calculated as follows.

(2) $$MAD_{i,j}=\frac{1}{M}\overset{M}{\underset{k=1}{\sum }}\left|h_{measured,k}-h_{DB}\left(\hat{\lambda }+i\Delta \lambda +p_{k,lon},\hat{\varphi }+j\Delta \varphi +p_{k,lat}\right)\right|\;\;\;=\frac{1}{M}\overset{M}{\underset{k=1}{\sum }}\left|\left(h_{baro,k}-\left|p_{k,alt}\right|\right)-h_{DB}\left(\hat{\lambda }+i\Delta \lambda +p_{k,lon},\hat{\varphi }+j\Delta \varphi +p_{k,lat}\right)\right|$$

where $p_{k,alt}=\;p_{k,u}$ is the vertical relative position to the closest point, $p_{k,\left(lon,lat\right)}=\left[p_{e}/R_{ew},p_{n}/R_{ns}\right]$ are the east and north direction relative position to the closest point, $R_{ew}$ is the Earth’s prime vertical radius of curvature, $R_{ns}$ is Earth’s meridional radius of curvature.

Comparing DEM and the relative position up to the closest point, correction values of −0.001° in latitude direction and +0.002° in longitude direction can be obtained. The correction values are the same as those of the ideal TRN with RA, shown in Figure 2.

**Figure 7:**
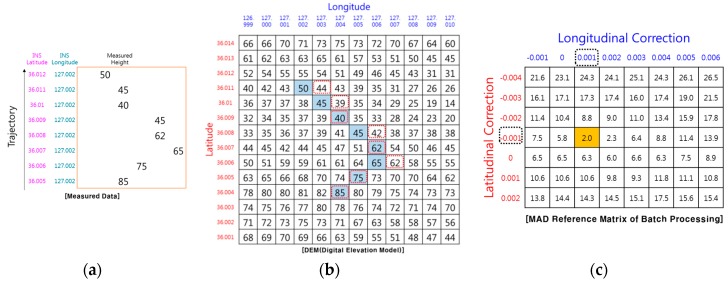
Example on TRN using miscalculated $p_{k}$: (**a**) Measured terrain profile; (**b**) DEM; (**c**) position correction value.

In order to perform TRN, it is necessary to calculate the relative position $p_{k}$ of the closest point. Even if the IRA outputs the correct range and angle to the TRN, if $p_{k}$ is miscalculated, the TRN calculates the wrong navigation solution. Figure 7 shows an example of TRN error caused by a miscalculated $p_{k,}$. It is assumed that there are no errors in $p_{k,alt}$ and $p_{k,lat}$, and that only $p_{k,lon}$ is calculated incorrectly. According to Equation (2), if there is an error of $p_{k,lon},$ the terrain database values at the position biased by the error are used for the MAD calculation. The errors of $p_{k,lon}$ are accumulated in the MAD calculation and cause the TRN longitude error. In this case, correction values of −0.001° in latitude direction and +0.001° in longitude direction can be obtained. The longitudinal correction value is different from the longitudinal correction value shown in Figure 6. Therefore, $p_{k}$ must be calculated accurately to obtain a correct TRN solution.

## 3. Relative Position Measurement Calculation Method Using IRA Output

### 3.1. Simple Method

According to existing research results, when roll and pitch angles are 0°, the relative position of the closest point can be calculated very simply by using the following formula [8].

(3) $$p=\left[\begin{array}{c} p_{e}\\ p_{n}\\ p_{u} \end{array}\right]=\left[\begin{array}{c} R\sin\theta \sin\emptyset \\ R\sin\theta \cos\emptyset \\ -R\cos\theta \end{array}\right]$$

where R is slant range, θ is look angle, and ∅ is heading angle.

Figure 5 shows the measurement model for IRA used in reference [8]. This calculation method is very intuitive and has the advantage of allowing the reader to understand how to calculate the relative position of the closest point. It is also very simple to calculate the relative position of the closest point in a TRN simulation that does not consider the influence of wind. This formula was useful for simulation development before flight testing.

The existing paper [8] does not show how to calculate the closest point when pitch exists, but that point can be calculated using the following formula.

(4) $$p=\left[\begin{array}{c} p_{e}\\ p_{n}\\ p_{u} \end{array}\right]=C_{b}^{n}\left(\gamma +\theta ,\vartheta ,-\psi \right)\left[\begin{array}{c} 0\\ 0\\ -R \end{array}\right]$$

where γ, ϑ, and ψ are roll, pitch and heading angle of the aircraft, respectively.

(5) $$C_{1}\left(\phi_{z}\right)=\;\left[\begin{array}{ccc} \cos\phi_{z} & \sin\phi_{z} & 0\\ -\sin\phi_{z} & \cos\phi_{z} & 0\\ 0 & 0 & 1 \end{array}\right]$$

(6) $$C_{2}\left(\phi_{x}\right)=\;\left[\begin{array}{ccc} 1 & 0 & 0\\ 0 & \cos\phi_{x} & \sin\phi_{x}\\ 0 & -\sin\phi_{x} & \cos\phi_{x} \end{array}\right]$$

(7) $$C_{3}\left(\phi_{y}\right)=\;\left[\begin{array}{ccc} \cos\phi_{y} & 0 & -\sin\phi_{y}\\ 0 & 1 & 0\\ \sin\phi_{y} & 0 & \cos\phi_{y} \end{array}\right]$$

(8) $$C_{b}^{n}\left(\phi_{z},\phi_{x},\phi_{y}\right)=\;C_{1}\left(\phi_{z}\right)C_{2}\left(\phi_{x}\right)C_{3}\left(\phi_{y}\right)$$

### 3.2. Conventional Method

In Section 2.2, the IRA output information about the closest points in the zero Doppler line. Before describing the formula for calculation of the relative position of the closest point, it is necessary to understand the zero Doppler line.

**Figure 8:**
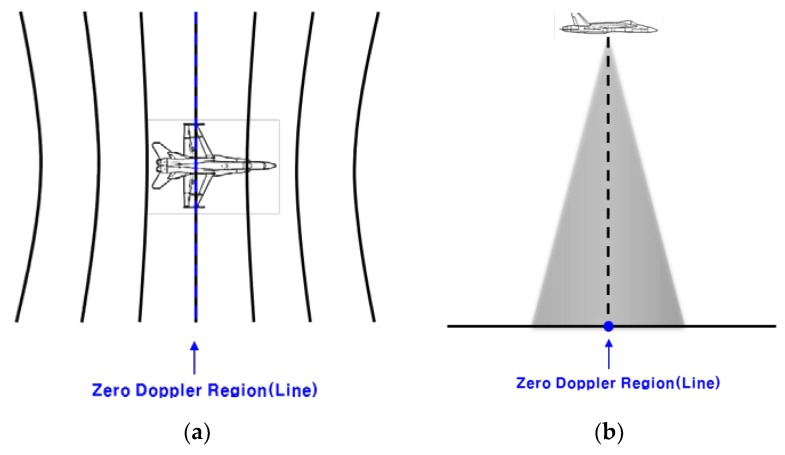
Isodop and zero Doppler region (line) of IRA on the aircraft: (**a**) top view; (**b**) side view.

To understand the zero Doppler line, it is important to first discuss the Doppler Effect. The Doppler Effect is caused by relative velocity between a source and an observer. For example, when a source is moving, if the observer is in front of the source, the wavelength becomes short and the frequency becomes high. If the observer is at the rear of the source, the wavelength becomes long and the frequency becomes low. The Doppler Effect also appears in the IRA on aircraft. When an aircraft is flying, the IRA transmits radio waves downward, and the Doppler radial velocity changes according to the relative distance from the aircraft to the point on the ground where the radio waves are received. The contour of constant radial velocity is called isodop, which is shown in Figure 8a. The zero Doppler line is a contour with a Doppler radial velocity of zero. The zero Doppler line is perpendicular to the velocity vector of the aircraft as shown in Figure 8b. 

**Figure 9:**
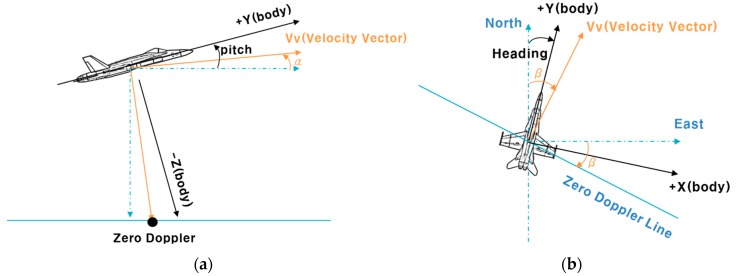
Comparison between aircraft’s attitude and velocity vectors in actual flight environment: (**a**) side view; (**b**) top view [12].

On the other hand, when we executed the flight test, we found that the attitude and velocity vectors of the aircraft are different. This is due to the angle of attack and the side slip angle, both caused by wind during flight. Figure 9 shows the difference between aircraft attitude and velocity vectors due to wind. Figure 9a is a side view of the aircraft and Figure 9b is a top view. Angle *α* is formed by the velocity vector of the aircraft with respect to the horizontal plane of the aircraft. Angle *β* is formed by the velocity vector of the aircraft with respect to the north axis. We will call these angles “effective observation angles.” In a simulation environment in which effects of wind are not considered, a zero Doppler line exists relative to the Euler angles (attitude) of the aircraft. However, in an actual flight environment in which the influence of wind exists, the effective observation angles (*α*, *β*), not the Euler angle, should be used to calculate the zero Doppler line. The effective observation angles *α* and *β* can be obtained using the following equation.

(9) $$\alpha =\tan^{-1}\frac{v_{u}}{\sqrt{{v_{e}}^{2}+{v_{n}}^{2}}}$$

(10) $$\beta =\{\begin{array}{c} \tan^{-1}\frac{v_{e}}{v_{n}}\;\;\;\;\;\;,\left|v_{n}\right|\geq \left|v_{e}\right|\\ \frac{\pi }{2}-\tan^{-1}\frac{v_{n}}{v_{e}}\;\;,\left|v_{n}\right|<\left|v_{e}\right| \end{array}$$

where $v_{e}$*,* $v_{n}$ and $v_{u}$ are the velocities measured by the inertial navigation system. 

The conventional formula used to calculate the relative position of the closest point using effective observation angles is presented in reference [11] as follows.

(11) $$p=\left[\begin{array}{c} p_{e}\\ p_{n}\\ p_{u} \end{array}\right]=\left[\begin{array}{c} R\cos\theta \sin\alpha \sin\beta +R\sin\theta \cos\beta \\ R\cos\theta \sin\alpha \cos\beta -R\sin\theta \sin\beta \\ -R\cos\theta \cos\alpha \end{array}\right]\;=R\left[\begin{array}{cc} \sin\alpha \sin\beta & \cos\beta \\ \sin\alpha \cos\beta & -\sin\beta \\ -\cos\alpha & 0 \end{array}\right]\left[\begin{array}{c} \cos\theta \\ \sin\theta \end{array}\right]=RS\left[\begin{array}{c} \cos\theta \\ \sin\theta \end{array}\right]$$

where

(12) $$S=\left[\begin{array}{cc} \sin\alpha \sin\beta & \cos\beta \\ \sin\alpha \cos\beta & -\sin\beta \\ -\cos\alpha & 0 \end{array}\right]$$

In the existing paper [11], if the roll angle (γ) is not 0°, (look angle (θ) + roll angle (γ)) is substituted for look angle (θ).

(13) $$p=\left[\begin{array}{c} R\cos\left(\theta +\gamma \right)\sin\alpha \sin\beta +R\sin\left(\theta +\gamma \right)\cos\beta \\ R\cos\left(\theta +\gamma \right)\sin\alpha \cos\beta -R\sin\left(\theta +\gamma \right)\sin\beta \\ -R\cos\left(\theta +\gamma \right)\cos\alpha \end{array}\right]$$

**Figure 10:**
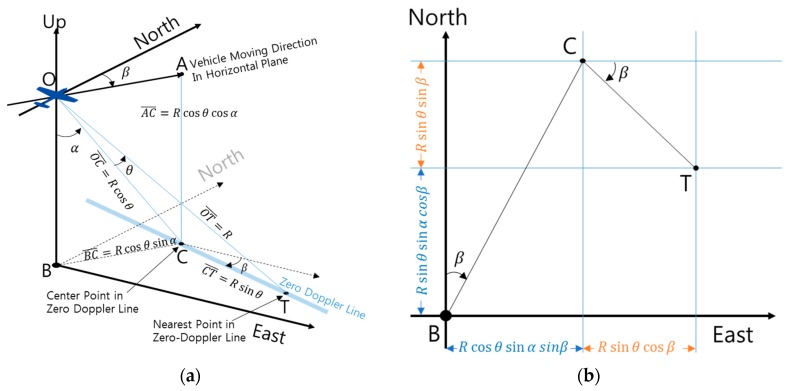
Relative position from aircraft to closest point: (**a**) 3-dimensional graph; (**b**) 2-dimensional graph [12].

The relative position p from the aircraft to the closest point is shown in Figure 10a. Figure 10b also shows the results of projecting Figure 10a onto the North-East plane.

### 3.3. Proposed Method

**Figure 11:**
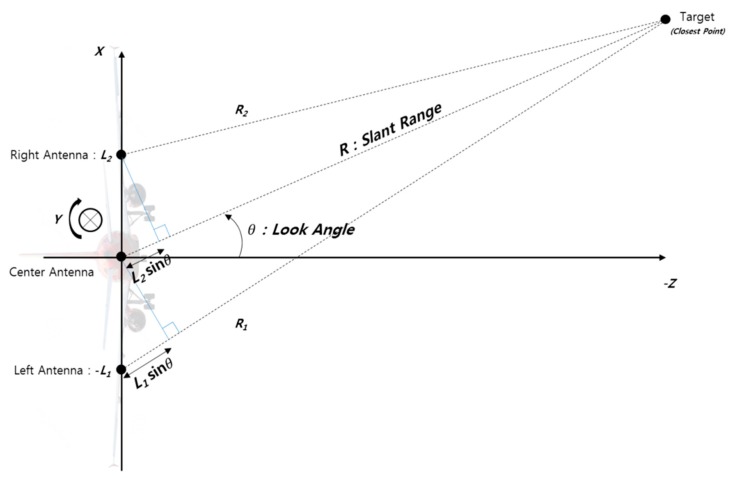
Look angle measurement of IRA [12].

Let’s see how IRA calculates and outputs the look angle (Figure 11). In the figure, the aircraft was assumed to be flying in a direction to penetrate the paper. The *X*-axis of the aircraft is the rightward direction of the aircraft (cross track), the *Y*-axis is the nose direction of the aircraft (along track, the direction penetrating the paper plane), and the *Z*-axis is the upward direction of the aircraft. The IRA requires two or more antennas, which are installed aligned with the *X*-axis (cross track) of the aircraft. The IRA measures the range from each antenna to the closest point (or target). The obtained range values are different, and the difference between the range values is used to calculate the phase difference. See reference [7] for details on how to compute the look angle using the phase difference between the slant ranges. The look angle is calculated using the difference of the phase. Since the IRA antenna is installed aligned with the *X*-axis of the aircraft, the look angle (*θ*) refers to the angle relative to the *X*-*Z* plane of the aircraft (−*Z* direction in the figure). In other words, the look angle is determined by the attitude of the flight vehicle, not by the velocity vector. On the other hand, the zero Doppler line is determined by the velocity vector rather than by the attitude of the aircraft. 

**Figure 12:**
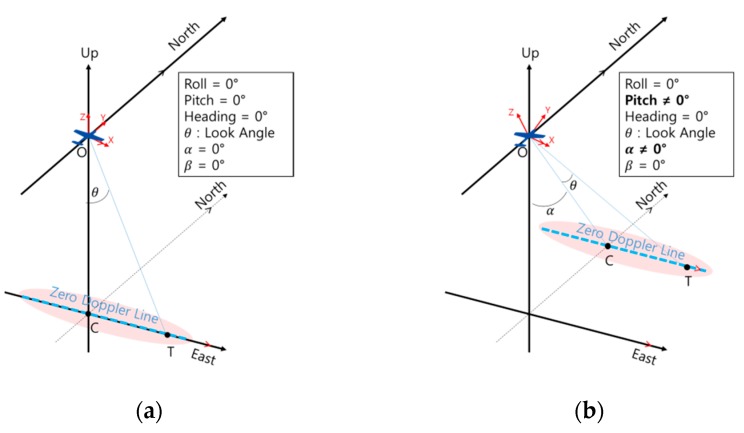

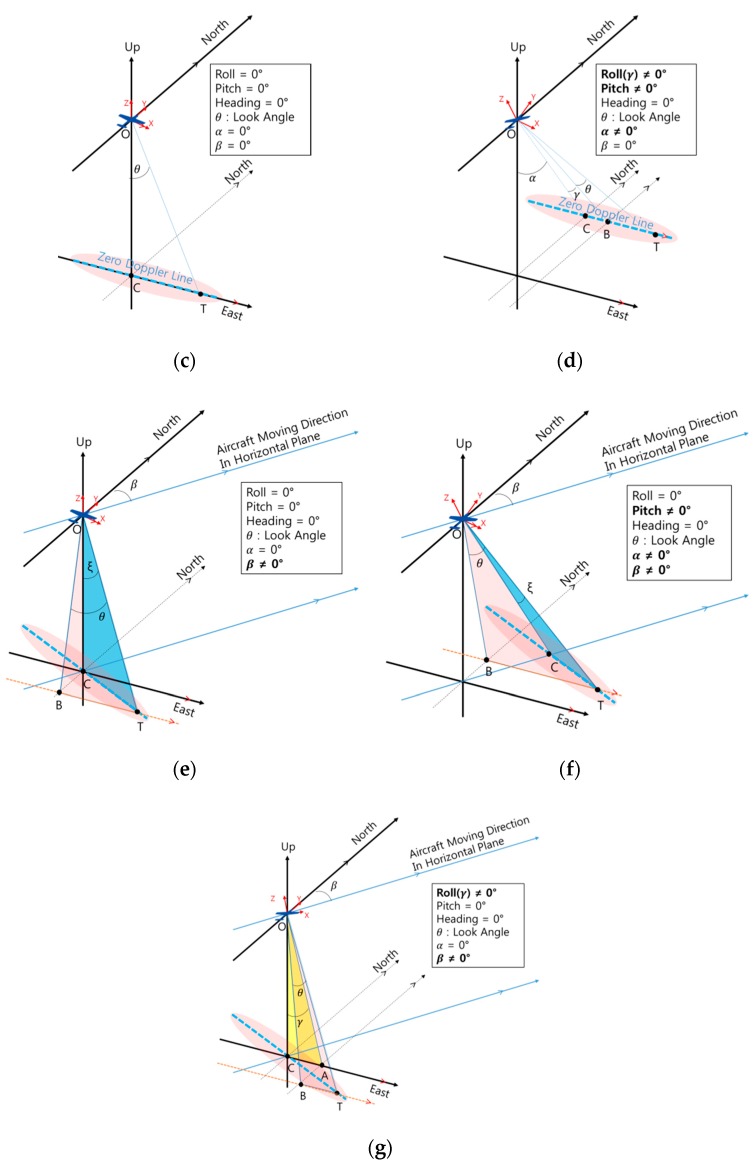
3-dimensional graph of relative position from aircraft to closest point in real flight condition: (**a**) case in which roll, pitch, heading, *α*, and *β* angles are all zero; (**b**) pitch and *α* angles are not zero; (**c**) roll angle is not zero; (**d**) roll, pitch and *α* angles are not zero; (**e**) *β* angle is not zero; (**f**) pitch, *α* and *β* angles are not zero; (**g**) roll and *β* angles are not zero.

To better understand where the zero Doppler line and closest points are located in a real flight environment, Figure 12 is attached. Figure 12a shows the environment in which the roll, pitch, heading, *α* (effective pitch), and *β* (effective heading) angles are all zero. In this case, the pitch and heading angles are the same for *α* and *β*, respectively, so that the zero Doppler line (the dotted blue line) is parallel to the *X*-axis of the flight, and the center of the zero Doppler line (point C) lies directly below the aircraft. In this case, the look angle is ∠COT, and can be used to find the location of the closest point. In addition to the condition of Figure 12a, Figure 12b shows a case in which the pitch angle and the *α* angle are not 0° due to wind blowing from the front. In this case, the center of the zero Doppler line moves forward by angle *α*, but the zero Doppler line is still parallel to the *X*-axis of the flight. The position of the closest point (point T) can be found by rotating *θ* at the center of the shifted zero Doppler line. Figure 12c,d illustrate the case in which the roll angle of the aircraft is not 0 under the conditions of Figure 12a,b. In both cases shown in Figure 12c,d, the zero Doppler line is parallel to the *X*-axis of the vehicle, even if the roll angle is not zero. Therefore, instead of using *θ* from Figure 12a,b, we can find the location of the closest point by using the value angle roll + *θ*. In addition to the conditions of Figure 12a,b, Figure 12e,f show cases in which the *β* angle is not 0 due to wind blowing from the side of the vehicle. In this case, the *X*-axis and zero Doppler lines of the aircraft are not parallel as shown in Figure 12a–d, but are in a twisted position. To find the closest point, we need the “effective look angle” ∠COT, expressed as angle *ξ*. However, the look angle is ∠BOT, which is not the same as ∠COT. In the case shown in Figure 12f, the center of the zero Doppler line is shifted by angle *α*. In this case, ∠COT is required to find the closest point. However, as shown in Figure 12e, because ∠COT is different from the look angle ∠BOT, we cannot use the look angle to find the closest point. As shown in Figure 12a–f, there is a difference between roll angle + look angle and *ξ* when there is wind blowing from the side. In addition to the condition of Figure 12e, Figure 12g shows a case in which the roll angle is not 0°. In this case, the roll angle is ∠COA and the look angle is ∠BOT. In the figure, we can see that the roll angle and the look angle are on different planes. It is not appropriate to add the roll angle and look angle arithmetically in this situation. 

In conclusion, to calculate the relative position from the IRA antenna to the closest point, an effective look angle *ξ* is required instead of an angle roll angle + look angle.

The formula for calculating *α* and *β* presented in the previous paper [11] was intuitively easy to derive. However, calculating the effective look angle, *ξ*, is not simple because this value must be obtained by synthesizing the velocity vector and the look angle calculated based on the attitude of the flight body. In this paper, we consider the above assumptions and derive the equation to calculate the effective look angle.

The position of the left antenna in the body frame is:

(14) $$x_{l}^{b}={\left[\begin{array}{ccc} -L_{1} & 0 & 0 \end{array}\right]}^{T}$$

The position of the right antenna in the body frame is:

(15) $$x_{r}^{b}={\left[\begin{array}{ccc} +L_{2} & 0 & 0 \end{array}\right]}^{T}$$

where

(16) $$x_{l}^{n}=\;C_{b}^{n}x_{l}^{b}\;$$

(17) $$x_{r}^{n}=\;C_{b}^{n}x_{r}^{b}$$

According to the IRA angle calculation algorithm (Figure 11), the range difference between the left antenna and the center antenna and the range difference between the right antenna and the center antenna are expressed as functions of the look angle (θ) and the antenna baseline (*L*_1_, $L_{2}$) as follows.

(18) $$R-\;\|x_{l}^{n}-p\|=L_{1}\sin\theta$$

(19) $$\|x_{r}^{n}-p\|-R=L_{2}\sin\theta$$

(20) $$\sqrt{{\left(x_{l}^{n}-p\right)}^{T}\left(x_{l}^{n}-p\right)}=R-L_{1}\sin\theta$$

(21) $$\sqrt{{\left(x_{r}^{n}-p\right)}^{T}\left(x_{r}^{n}-p\right)}=R+L_{2}\sin\theta$$

The squares of both sides of Equations (20) and (21) are as follows,

(22) $$L_{1}^{2}-2x_{l}^{n^{T}}p+R^{2}=R^{2}-2L_{1}R\sin\theta +L_{1}^{2}\sin^{2}\theta$$

(23) $$L_{2}^{2}-2x_{r}^{n^{T}}p+R^{2}=R^{2}+2L_{2}R\sin\theta +L_{2}^{2}\sin^{2}\theta$$

After removing $R^{2}$ from both sides and dividing by −2R,

(24) $$\frac{L_{1}^{2}}{-2R}+\frac{1}{R}x_{l}^{n^{T}}p=L_{1}\sin\theta -\frac{L_{1}^{2}\sin^{2}\theta }{2R}$$

(25) $$\frac{L_{1}^{2}}{-2R}+\frac{1}{R}x_{r}^{n^{T}}p=-L_{2}\sin\theta -\frac{L_{1}^{2}\sin^{2}\theta }{2R}$$

Equations (24) and (25) are approximated as follows by $L_{1}\ll R,\;L_{2}\ll R$

(26) $$\frac{1}{R}x_{l}^{n^{T}}p=L_{1}\sin\theta$$

(27) $$\frac{1}{R}x_{r}^{n^{T}}p=-L_{2}\sin\theta .\;$$

Substituting Equations (14)–(17) into the above two equations,

(28) $$\frac{1}{R}x_{l}^{n^{T}}p=\frac{1}{R}L_{1}\left[\begin{array}{ccc} -1 & 0 & 0 \end{array}\right]C_{n}^{b}RS\left[\begin{array}{c} \cos\xi \\ \sin\xi \end{array}\right]=L_{1}\left[\begin{array}{ccc} -1 & 0 & 0 \end{array}\right]C_{n}^{b}S\left[\begin{array}{c} \cos\xi \\ \sin\xi \end{array}\right]=L_{1}\sin\theta .\;$$

(29) $$\frac{1}{R}x_{r}^{n^{T}}p=\frac{1}{R}L_{2}\left[\begin{array}{ccc} +1 & 0 & 0 \end{array}\right]C_{n}^{b}RS\left[\begin{array}{c} \cos\xi \\ \sin\xi \end{array}\right]\;=L_{2}\left[\begin{array}{ccc} +1 & 0 & 0 \end{array}\right]C_{n}^{b}S\left[\begin{array}{c} \cos\xi \\ \sin\xi \end{array}\right]=-L_{2}\sin\theta$$

where $p=R\left[\begin{array}{cc} \sin\alpha \sin\beta & \cos\beta \\ \sin\alpha \cos\beta & -\sin\beta \\ -\cos\alpha & 0 \end{array}\right]\left[\begin{array}{c} \cos\xi \\ \sin\xi \end{array}\right]=RS\left[\begin{array}{c} \cos\xi \\ \sin\xi \end{array}\right]$, as shown in Equation (11). 

Therefore, if both sides of Equations (28) and (29) are divided into $L_{1}$ and $L_{2}$, respectively, the following single expression for ξ is acquired.

(30) $$\left[\begin{array}{ccc} -1 & 0 & 0 \end{array}\right]C_{n}^{b}S\left[\begin{array}{c} \cos\xi \\ \sin\xi \end{array}\right]=\left[\begin{array}{cc} t_{1} & t_{2} \end{array}\right]\left[\begin{array}{c} \cos\xi \\ \sin\xi \end{array}\right]=\sin\theta$$

where $t_{1}\;and\;t_{2}$ are calculated as follows,

(31) $$\left[\begin{array}{cc} t_{1} & t_{2} \end{array}\right]=\left[\begin{array}{ccc} -1 & 0 & 0 \end{array}\right]C_{n}^{b}S=\left[\begin{array}{ccc} -1 & 0 & 0 \end{array}\right]C_{b}^{n^{T}}S\;$$

where $C_{b}^{n}$ is a transformation matrix from the body frame to the navigation frame, shown in Equation (8), $S=\left[\begin{array}{cc} \sin\alpha \sin\beta & \cos\beta \\ \sin\alpha \cos\beta & -\sin\beta \\ -\cos\alpha & 0 \end{array}\right]$, as shown in Equation (12).

Dividing both sides by $\sqrt{t_{1}^{2}+t_{2}^{2}}$ we obtain,

(32) $$\frac{t_{1}}{\sqrt{t_{1}^{2}+t_{2}^{2}}}\cos\xi +\frac{t_{2}}{\sqrt{t_{1}^{2}+t_{2}^{2}}}\sin\xi =\frac{\sin\theta }{\sqrt{t_{1}^{2}+t_{2}^{2}}}$$

Substituting sinη for $\frac{t_{1}}{\sqrt{t_{1}^{2}+t_{2}^{2}}}$ and cosη for $\frac{t_{2}}{\sqrt{t_{1}^{2}+t_{2}^{2}}}$ we obtain,

(33) $$\sin\eta \cos\xi +\cos\eta \sin\xi =\sin\left(\eta +\xi \right)=\frac{\sin\theta }{\sqrt{t_{1}^{2}+t_{2}^{2}}}$$

(34) $$\eta +\xi =\sin^{-1}\frac{\sin\theta }{\sqrt{t_{1}^{2}+t_{2}^{2}}}$$

(35) $$\xi =\sin^{-1}\frac{\sin\theta }{\sqrt{t_{1}^{2}+t_{2}^{2}}}-\eta ={\;\sin}^{-1}\frac{\sin\theta }{\sqrt{t_{1}^{2}+t_{2}^{2}}}-\tan^{-1}\frac{t_{1}}{t_{2}}$$

The relative position of the closest point can be calculated using the derived effective look angle ξ as follows.

(36) $$p=\left[\begin{array}{c} R\cos\xi \sin\alpha \sin\beta +R\sin\xi \cos\beta \\ R\cos\xi \sin\alpha \cos\beta -R\sin\xi \sin\beta \\ -R\cos\xi \cos\alpha \end{array}\right]$$

## 4. Performance Evaluation

### 4.1. Flight Test Preparation

TRN simulation is a good method to verify that the proposed formula actually calculates the position of the closest point accurately. However, the proposed formula is effective in dynamic environments with wind influences. Therefore, we carried out an actual flight test to obtain the real data needed for TRN simulation.

**Figure 13:**
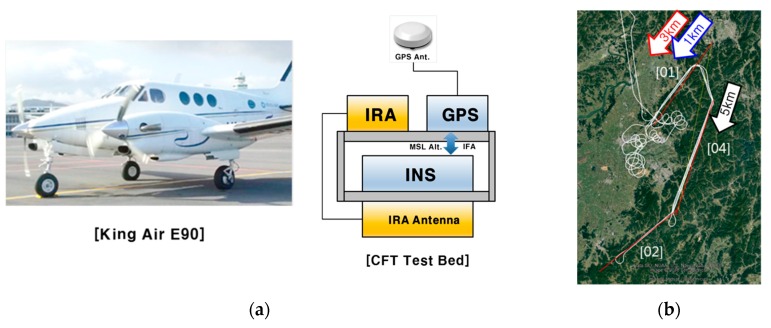
Flight test preparation (**a**) System configuration; (**b**) Trajectory [12].

The configuration for the flight test was as follows. The IRA used for the flight test was developed by the Agency for Defense Development and Hanwha Systems as described in Section 1 and Reference [13]. The aircraft used in the test was a King Air 60 model, on which the test fixture was mounted. A total of three IRA antennas were installed at the bottom of the mounting plate. The Inertial Navigation System (INS) was installed on the opposite side of the mounting plate where the IRA antenna is installed (Figure 13a). Since the machining error of the test fixture is negligibly small, it was assumed that the INS and IRA antennas were aligned without mounting error. The navigation system consists of a navigation grade INS and Global Positioning System (GPS). INS was designed to compensate for position, velocity, and attitude errors by using GPS to perform “In Flight Alignment (IFA)” during navigation. The altitude of the INS was corrected based on the GPS altitude (Mean Sea Level). When performing a real TRN, the altitude of the INS was corrected based on the barometric altimeter. The altitude error of barometric altimeter varies depending on the altitude or temperature of flight area, and is generally larger than the altitude error of GPS. Therefore, GPS correction was applied instead of the barometric altimeter. The Carrier Differential Global Positioning System (CDGPS) solution was used to more accurately correct the position error of the aircraft after the flight test. GPS signals were distributed from GPS antennas originally mounted on the aircraft. The GPS antenna was installed 2 m above the IRA antenna at the top of the aircraft, which was compensated for in the performance evaluation. The flight trajectory was selected for mountainous areas (Figure 13b) Table 1 shows the information on the flight test trajectory.

In conclusion, for accurate performance evaluation, errors other than IRA were rejected as much as possible.

### 4.2. Flight Test Data Analysis

We successfully obtained the real data of the INS, the GPS and the IRA from the flight test. All the real data were time synchronized using GPS Time. We verified that wind effect actually existed in the data and that the positions of the closest points calculated using each formula are different.

**Figure 14:**
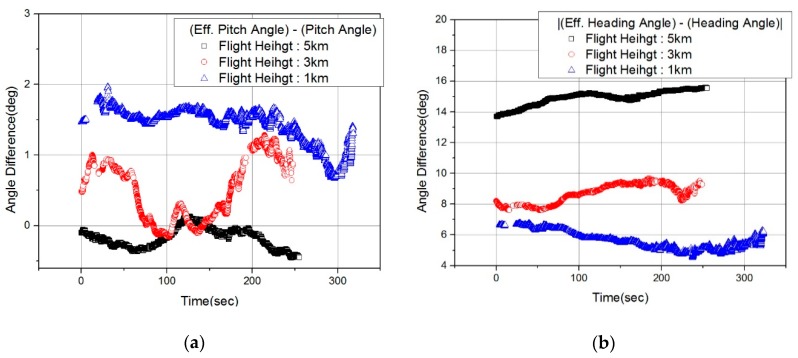
Comparison between aircraft attitude and effective observation angle: (**a**) Pitch angle difference; (**b**) Heading angle difference.

**Figure 15:**
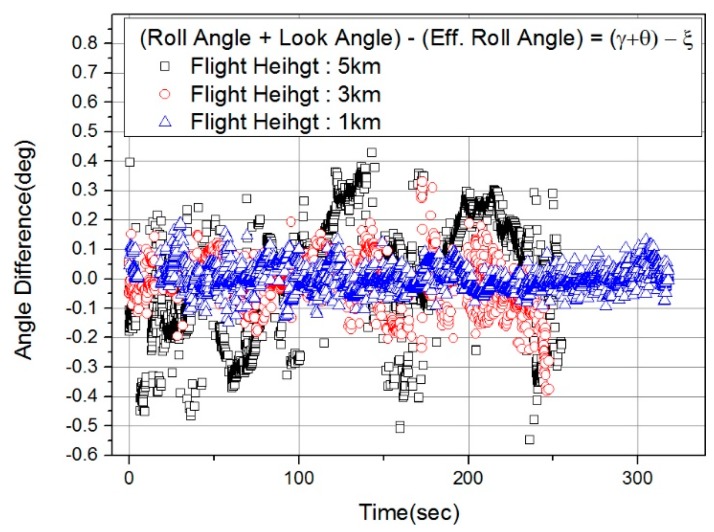
Difference between angles (*γ* + *θ*) and *ξ.*

First, in order to check whether the wind effect was present in the actual flight test results, the effective pitch and heading angle were compared with the pitch and heading of the flight body (Figure 14). The effective pitch angle did not show a large difference by altitude, but a clear difference between effective pitch angle and pitch angle can be seen in Figure 14a. It cannot be determined whether the heading angle difference depends on the trajectory or on the altitude, but the heading angle difference increases in the order of 1 km, 3 km and 5 km (Figure 14b). The cause for this is not clear, but the results show that the difference between *β* (effective heading angle) and heading angle was greatest at flight altitude of 5 km, which means the effect of winds blowing from the sides of the aircraft was strongest. In addition, we have argued that there is a difference between the roll angle + look angle (∠COA + ∠BOT in Figure 12g) and *ξ* (effective roll angle) due to the *β* value, as described in Section 3.3. Figure 15 shows the difference between roll angle + look angle and *ξ*. As expected, the maximum difference between the roll angles was largest at a flight altitude of 5 km, when the *β* value was largest. 

Second, we checked that the positions of the closest points calculated using each formula were different. The simple method (Equation (3) in Section 3.1) has been shown to be significantly less accurate than the conventional method (Equation (13) in Section 3.2) from the previous paper [12], and its results are excluded from the results analysis in this paper. The proposed method (Equation (36) in Section 3.3) is compared with the conventional method. The horizontal position difference between the closest points found using each method is defined as follows.

(37) $$\Delta p_{k,e}=p_{k,e\left(proposed\;method\right)}-\;p_{k,e\left(conventional\;method\right)}$$

(38) $$\Delta p_{k,n}=p_{k,n\left(proposed\;method\right)}-\;p_{k,n\left(conventional\;method\right)}$$

(39) $$\Delta p_{k}=\sqrt{\Delta {p_{k,e}}^{2}+\;\Delta {p_{k,n}}^{2}}$$

where $p_{k,e}$ and $p_{k,n}$ are the east and north direction relative distance to the closest point, $p_{k\left(proposed\;method\right)}$ and $p_{k\left(conventional\;method\right)}$ are the relative distance to the closest point calculated using the proposed method and the conventional method.

**Figure 16:**
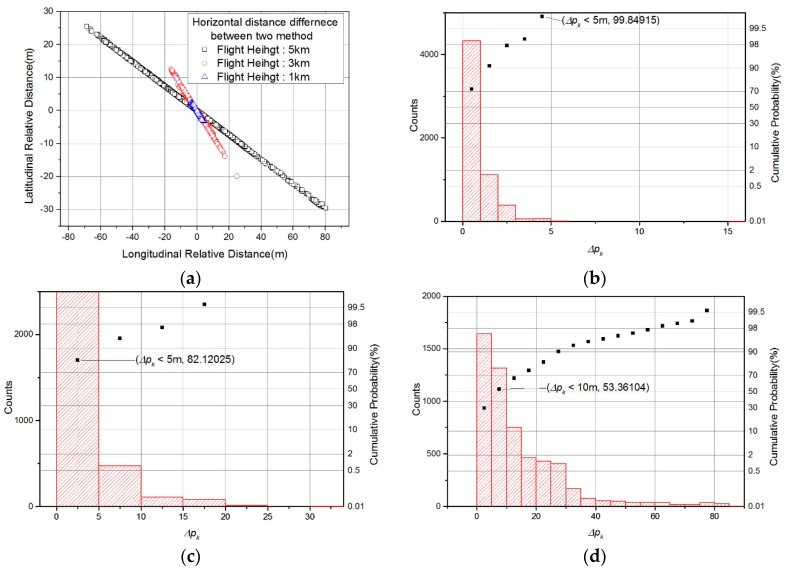
Horizontal position difference between closest points obtained using conventional method and proposed method: (**a**) horizontal graph; (**b**) histogram and cumulative probability at flight altitudes of 1 km; (**c**) 3 km; (**d**) 5 km.

Figure 16 shows $\Delta p_{k}$ obtained using both calculation methods. Figure 16a shows that the maximum value of $\Delta p_{k}$ was largest at the flight altitude of 5 km and smallest at the flight altitude of 1 km. Figure 16b–d show histograms and cumulative probabilities of $\Delta p_{k}$ at each altitude. At a flight altitude of 1km, 99% of $\Delta p_{k}$ were within 5 m. At a flight altitude of 3 km, 85.6% of $\Delta p_{k}$ were less than 5 m. The resolution of the DEM used in the TRN simulation was about 10 m, but $\Delta p_{k}$ of less than 5 m is too small to confirm the performance difference between the two formulas. At a flight altitude of 5 km, 53.4% of $\Delta p_{k}$ were less than 10 m. 

The horizontal position difference between the two formulas can be roughly calculated by the following equation.

(40) $$\Delta p_{k}≒\;R\tan(\left(\theta +\gamma \right)-\;\xi )$$

where *R* is slant range of IRA, θ is look angle of IRA, γ is roll angle of INS and ξ is effective look angle.

Since the range (*R*) and angle difference ((θ+γ)− ξ) were largest when the flight altitude was 5 km, the maximum value of $\Delta p_{k}$ was also largest for the Equation (40). Therefore, we performed the TRN simulation using the data at the flight altitude of 5 km and compared the performance of each formula.

### 4.3. TRN Simulation Conditions

**Figure 17:**
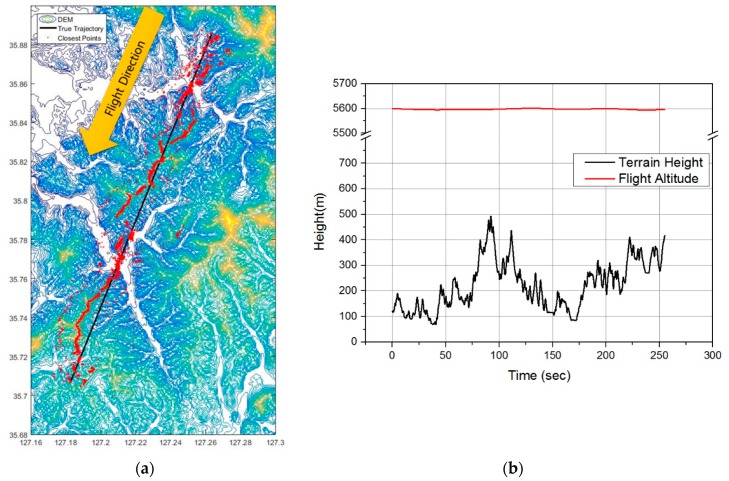
Simulation trajectory: (**a**) horizontal flight trajectory; (**b**) flight altitude and terrain height profile.

In order to evaluate the performance of the proposed equation, TRN simulation was performed under the following conditions. CDGPS corrected position, GPS corrected velocity and attitude data were used as true data for TRN simulation. The simulation trajectory was the same as the 5 km flight test trajectory. The flight altitude and terrain height profile for the trajectory is shown in Figure 17. The average flight height was 5600 m which was about 5100 m higher than the maximum elevation of the terrain profile. The average altitude of the terrain profile was 223 m, and the standard deviation was 95 m. The IRA data obtained from the flight test were used for TRN simulations. The closest points calculated using the IRA output are marked in red (Figure 16a), and it seems that the IRA usually measured the points of the mountain peaks or ridges. For the TRN simulation, the Batch Processing algorithm introduced in Section 2 was used. The 13-state Extended Kalman Filter (EKF) based INS / TRN correction filter was applied to compensate for the velocity error of the INS. The velocity error estimated by the INS / TRN correction filter was used for TRN input compensation. Detailed conditions of the simulation are shown in Table 2. All of the other parameters were the same except for the formula for calculation of the relative position of the closest point.

TRN simulations were performed for four cases to compare the performance difference according to $\Delta p_{k}$, and the details of the simulation are shown in Table 3. The Monte Carlo (MC) simulation was performed 50 times for statistical error comparisons.

### 4.4. TRN Simulation Results

**Figure 18:**
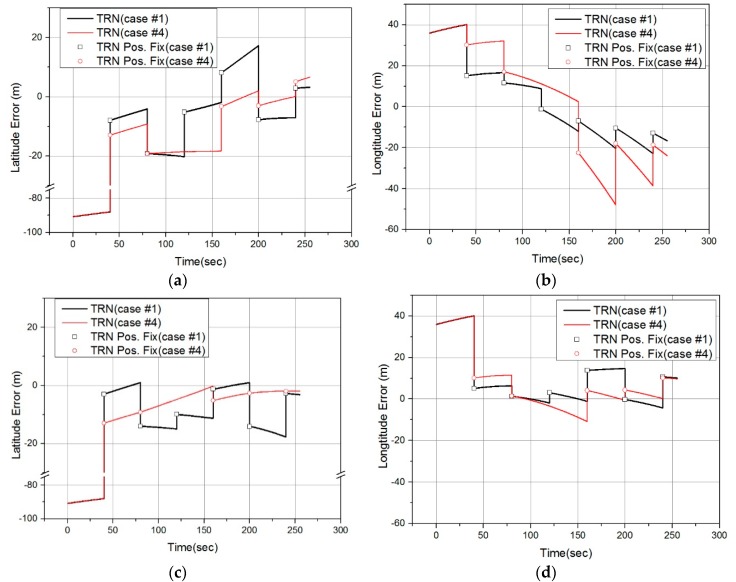
TRN simulation result graphs: (**a**) latitude error for Conventional method; (**b**) longitude error for Conventional method; (**c**) latitude error for Proposed method; (**d**) longitude error for Proposed method.

Figure 18 shows latitude and longitude error graphs for TRN simulation case #1 and case #4. These graphs show 12th simulation result among the 50 times MC simulations. Each graph shows the position error of batch processing. The position fix was performed every 40 s, and the position error at that time was indicated in each graph. The performance difference between case #1 and case #4 is not clearly seen in Figure 18a,c,d. On the other hand, the position error of case #4 was always larger than the position error of case #1 as shown in Figure 18b. We argued that the horizontal error of $p_{k}$ causes the horizontal error of TRN in Section 2.2. In Figure 16, since $\Delta p_{k,e}$ was larger than $\Delta p_{k,n}$, the longitude error was relatively larger than the latitude error in the simulation result of the conventional method. 

**Figure 19:**
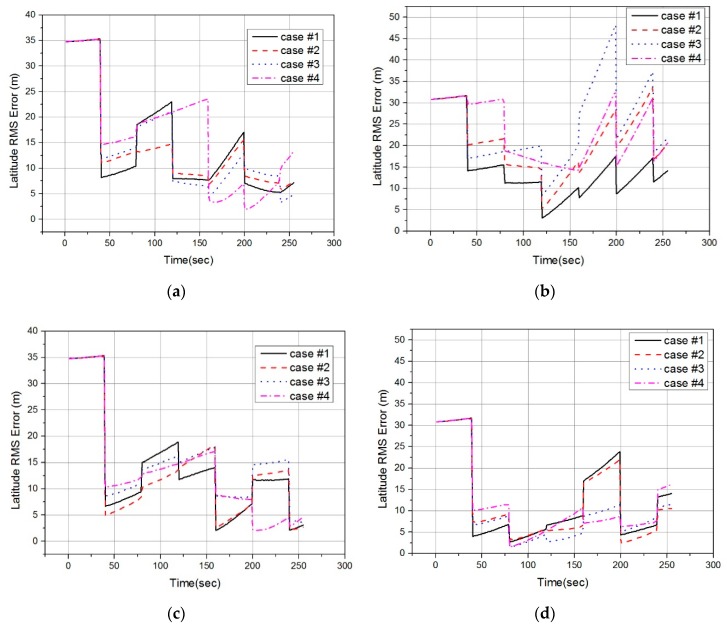
Monte Carlo simulation result graphs: (**a**) latitude RMS error for Conventional method; (**b**) longitude RMS error for Conventional method; (**c**) latitude RMS error for Proposed method; (**d**) longitude RMS error for Proposed method.

Figure 19 compares latitude and longitude RMS errors obtained from the 50 times MC TRN simulation. Likewise, the longitude error of case #1 is smaller than that of case #2–#4 in the entire simulation of the conventional method as shown in Figure 19b. However, the performance difference between cases #1 to #4 of the proposed method is not clearly seen, as shown in Figure 19c,d.

Table 4 shows the average latitude and longitude RMS errors for each TRN simulation case. The average RMS errors were calculated using the RMS errors at the time of the TRN position fix in Figure 19. In other words, data in the propagation period without the TRN position fix was excluded when calculating the average RMS errors. In the simulation result of the conventional method, the larger $\Delta p_{k}$, the larger position RMS error. In the simulation result of the proposed method, the performance difference according to $\Delta p_{k}$ is not clearly seen. In the simulation case #1, there wasn’t a significant difference of position error between two methods. In the simulation cases 2–4, the position errors of the proposed method were smaller than that of the conventional method. From the results of the TRN simulation, it can be concluded that the proposed method calculates the closest point more accurately than the conventional method. The TRN performance is degraded by calculating the closest point using the conventional method at high altitude where the wind blowing from the side is strong. However, the proposed method does not degrade the TRN performance because it calculates the accurate position of the closest point even in high altitude and windy environments.

## 5. Conclusions

In this paper, we proposed a new formula using IRA output to calculate the relative position of the closest point. Since the attitude and velocity vector of aircraft are different due to the wind when the aircraft is actually flying, the existing relative position calculation method using attitude becomes inaccurate. We derived the “effective look angle” equation using IRA’s look angle calculation principle and proposed a formula to calculate the relative position of the closest terrain point by using the velocity vector of the aircraft and the effective look angle. The proposed formula was verified with real data from actual flight. The flight test results show that the positions of the closest points calculated using the conventional method and the proposed method are different because of wind effect. The difference of the relative position of the closest point calculated using each equation was largest at the flight height of 5 km which had largest influence of wind blowing from the side. The accuracy of the proposed formula and conventional formula were evaluated by TRN simulation. The TRN simulation results indicate that the proposed formula calculates the closest points more accurately than the conventional formula. Using the proposed method, the performance degradation of TRN can be prevented even in high altitude and windy environments.

## Figures and Tables

**Table 1:** Information on flight trajectory.

Trajectory (km)	Average Flight Height (m)	Average Terrain Elevation (m)	Flight Time (s)	Flight Distance (km)	Average Flight Velocity (m/s)
1	1560	140	318	27	86
3	3640	130	248	21	85
5	5600	220	255	21	83

**Table 2:** Simulation condition.

Object	Parameters	Value
Accelerometer	Bias (1σ)	50 μg
	Random Walk (1σ)	10 μg
Gyroscope	Bias (1σ)	0.005 °/h
	Random Walk (1σ)	0.005 °/$\sqrt{h}$
Initial Position Error	Latitude, Longitude, Height (1σ)	30 m, 30 m, 15 m
Initial Velocity Error	E, N, U (1σ)	0.1 m/s
Initial attitude Error	Roll, Pitch, Heading (1σ)	0.05 mrad, 0.05 mrad, 1 mrad
Batch Processing TRN	Resolution of Ref. Matrix	5 m
	Size of Ref. Matrix	101 × 101
	Correlation Algorithm	MAD
	Position Fix Period	40 s
DEM	Resolution	0.3 arcsec, (≒ 10 m)
IRA	Output Frequency	50 Hz
INS / TRN CF	Execution cycle	50 Hz
	Measurement noise (R)	(10 m)^2^

**Table 3:** Simulation cases.

	Case #1	Case #2	Case #3	Case #4
IRA output used in simulation	All Data	$\Delta p_{k}$> 5 m	$\Delta p_{k}$> 10 m	$\Delta p_{k}$> 15 m

**Table 4:** Simulation results.

Calculation Method	Error	Average RMS Error (m)			
		Case #1	Case #2	Case #3	Case #4
Conventional method	Latitude Error	10.08	9.35	10.43	12.61
	Longitude Error	10.02	15.92	19.46	21.69
	Position Error	14.21	18.46	22.08	25.09
Proposed method	Latitude Error	9.60	9.11	11.46	10.05
	Longitude Error	9.58	8.91	6.73	9.64
	Position Error	13.56	12.74	13.29	13.93

## References

[1] Siouris G.M. (2004). Missile Guidance and Control Systems.

[2] Taurus Systems Gmh www.taurus-systems.de.

[3] Groves P.D. (2013). Principles of GNSS, Inertial, and Multisensor Integrated Navigation Systems.

[4] Raney R.K. (1998). The delay/Doppler radar altimeter. IEEE Trans. Geosci. Remote Sens..

[5] Honeywell. https://aerocontent.honeywell.com/aero/common/documents/myaerospacecatalog-documents/MilitaryAC/PTAN.pdf.

[6] Rosen P.A., Hensley S., Joughin I.R., Li F.K., Madsen S.N., Rodriguez E., Goldstein R.M. Synthetic aperture radar interferometry. https://trs.jpl.nasa.gov/handle/2014/20793.

[7] Lee D.T., Jung H.S., Yoon G.W. (2011). An Efficient Interferometric Radar Altimeter (IRA) Signal Processing to Extract Precise Three-dimensional Ground Coordinates. Korean J. Remote Sens..

[8] Jeong S.-H., Yoon J.H., Park M.-G., Kim D.-Y., Sung C.-K., Kim H.-S., Kim Y.-H., Kwak H.-J., Sun W., Yoon K.J. (2012). A performance analysis of terrain-aided navigation (TAN) algorithms using interferometric radar altimeter. J. Korean Soc. Aeronaut. Space Sci..

[9] Kim H.S., Sung C.K., Yoo K.J. Simulation for the TRN using Interferometric Radar Altimeter and PMF. Proceedings of the 2011 KSAS Spring Conference.

[10] Kim Y., Park J., Bang H. (2018). Terrain-Referenced Navigation using an Interferometric Radar Altimeter. Navig. J. Inst. Navig..

[11] Sung C.K., Nam S.H., Yu M.J. Terrain Referenced Navigation Based on Robust Point Mass Filter Using Variance Adjusted Discrete Normal PDF and Mean Valued Likelihood. Proceedings of the ION 2017 Pacific PNT Meeting.

[12] Oh J., Sung C.K., Lee J.S., Yu M.J. A new method to calculate relative distance of closest terrain point using interferometric radar altimeter output in real flight environment. Proceedings of the 2018 IEEE/ION Position, Location and Navigation Symposium (PLANS).

[13] Paek I., Lee S., Yoo K., Jang J.H. (2015). An Implementation of Interferometric Radar Altimeter Simulator. J. Korean Inst. Electromagn. Eng. Sci..

